# Supplementary material for: Direct-laser writing for subnanometer focusing and single-molecule imaging
Source: Nat Commun. 2022 Feb 3;13:647. doi: 10.1038/s41467-022-28219-6 (PMC8813935; doi:10.1038/s41467-022-28219-6)
Supplement: Supplementary file 1 — Supplementary information [file 41467_2022_28219_MOESM1_ESM.pdf]

Supplementary information

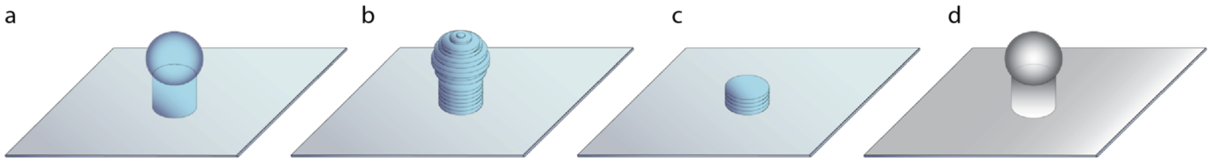

Supplementary Figure 1: Fundamental steps for the fabrication of 3D fiducials. The workflow used to produce fiducials. **a,** first a 3D CAD model is developed to illustrate the ideal fiducial. **b**, Next the designed model is converted into sectioned layers as the fabrication is performed in a layer-by-layer fashion. **c**, a partially produced fiducial illustrating the fabrication underway. **d**, the final fiducial produced.

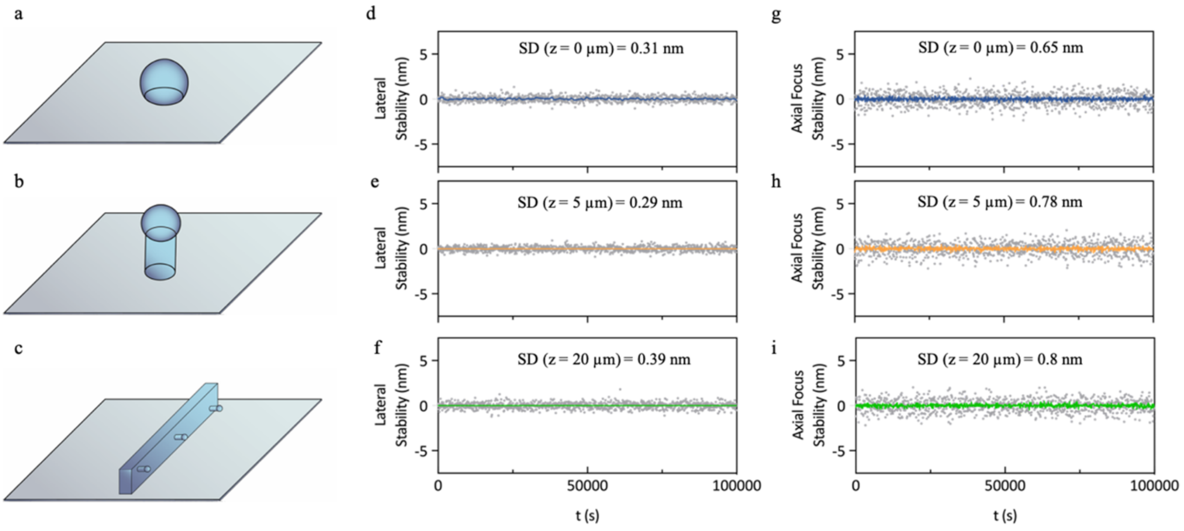

Supplementary Figure 2: Long-term focus-locking graphs as a function of time. Focus-locking was performed for on 3 different axial planes for ~ 1 day. **a-c**, Schematics of 3D nanoprinted fiducials. **d-f**, Lateral (x/y) stability with a standard deviation of **d**, 0.31 nm at the surface; **e**, 0.29 nm at a 5 µm depth and **f,** 0.39 nm at a 20 µm depth. **g-i**, Subnanometer focusing with a standard deviation of **g**, 0.65 nm at the surface; **h**, 0.78 nm at a 5 µm depth and **i**, 0.8 nm at a 20 µm depth. For **d-i**; grey symbols represent stability and the colored line represents a 10-point average (1/2000 points plotted).

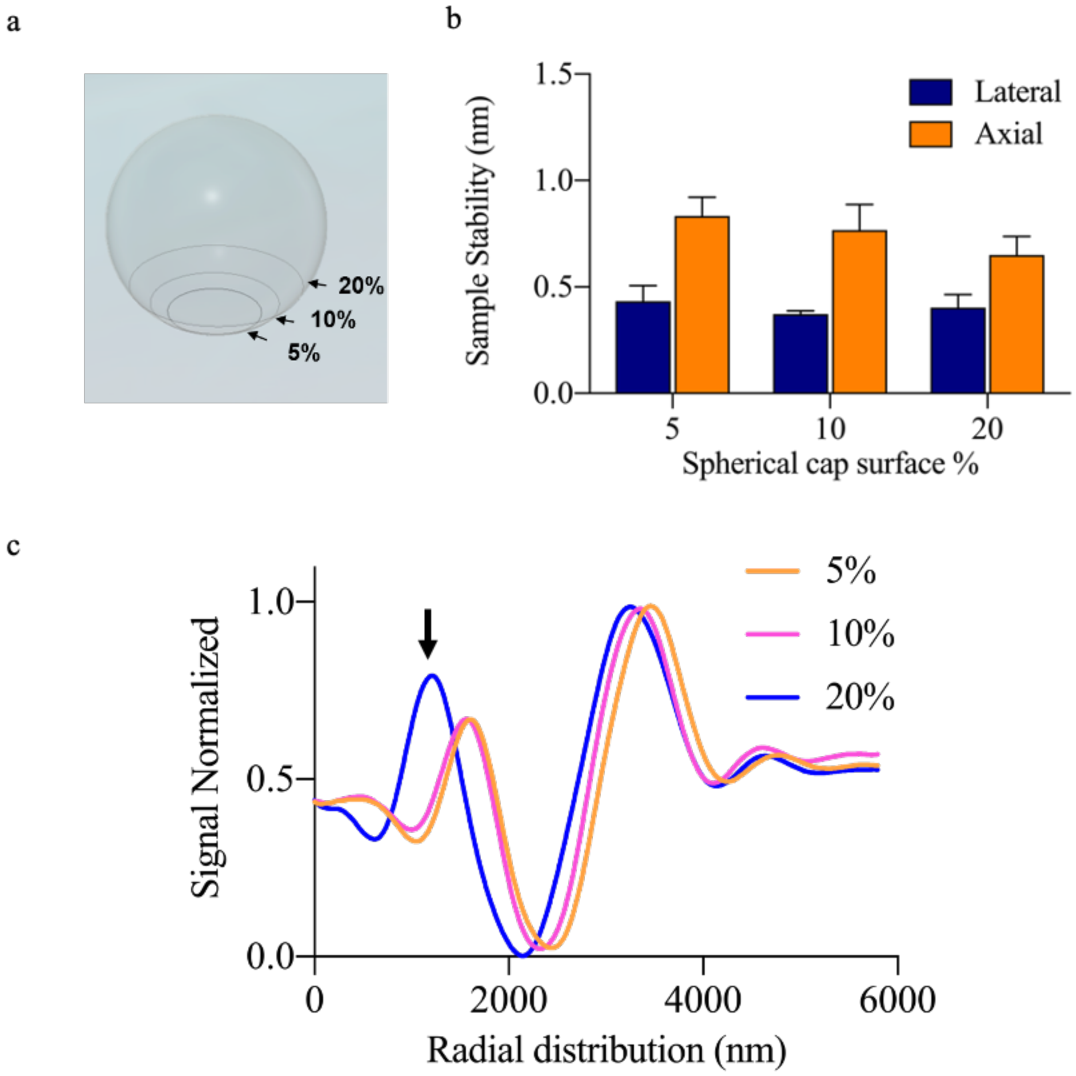

Supplementary Figure 3: Influence of glass-polymer interface and 3D focus lock. a, Illustration showing the contours of three different spherical caps on a 3 µm diameter spherical fiducial. **b**, 3D focus lock improvement as a function of spherical cap of 5%, 10% and 20% (n = 50 fiducials). Data are presented as mean values and error bars are the standard deviations. **c**. Comparison of the normalized radial intensity profile at z = 0. Improved contrast for a spherical cap of 20% (blue line) is indicated by the black arrow (n_{media} = 1.33). The signal variation stems from the range of incident/exit angles of the brightfield illumination at glass-polymer interface

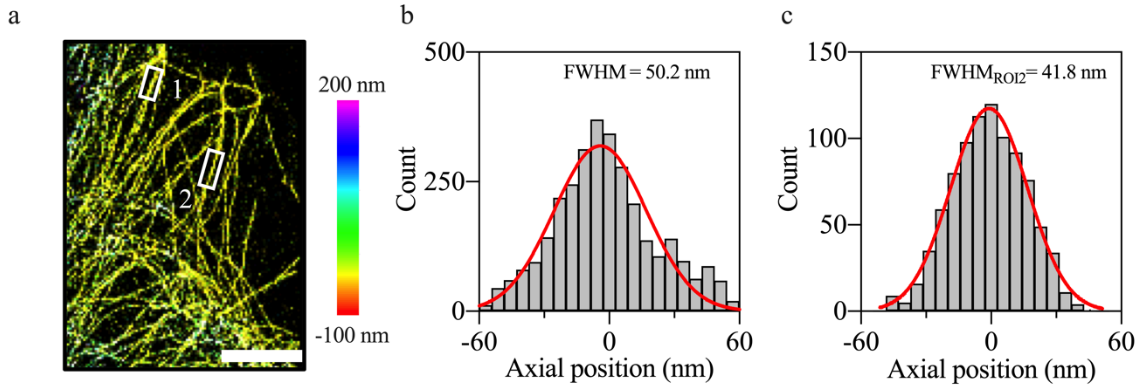

Supplementary Figure 4: Microtubule axial profiles. **a**, Close-up of a three-dimensional super-resolved image of microtubules shown in Fig 3c (n =12). Scale bar = 5 µm. **b and c**, Axial profiles of microtubules highlighted by the square regions with resolutions of 50.2 (**b**) and 41.8 nm (**c**), respectively.

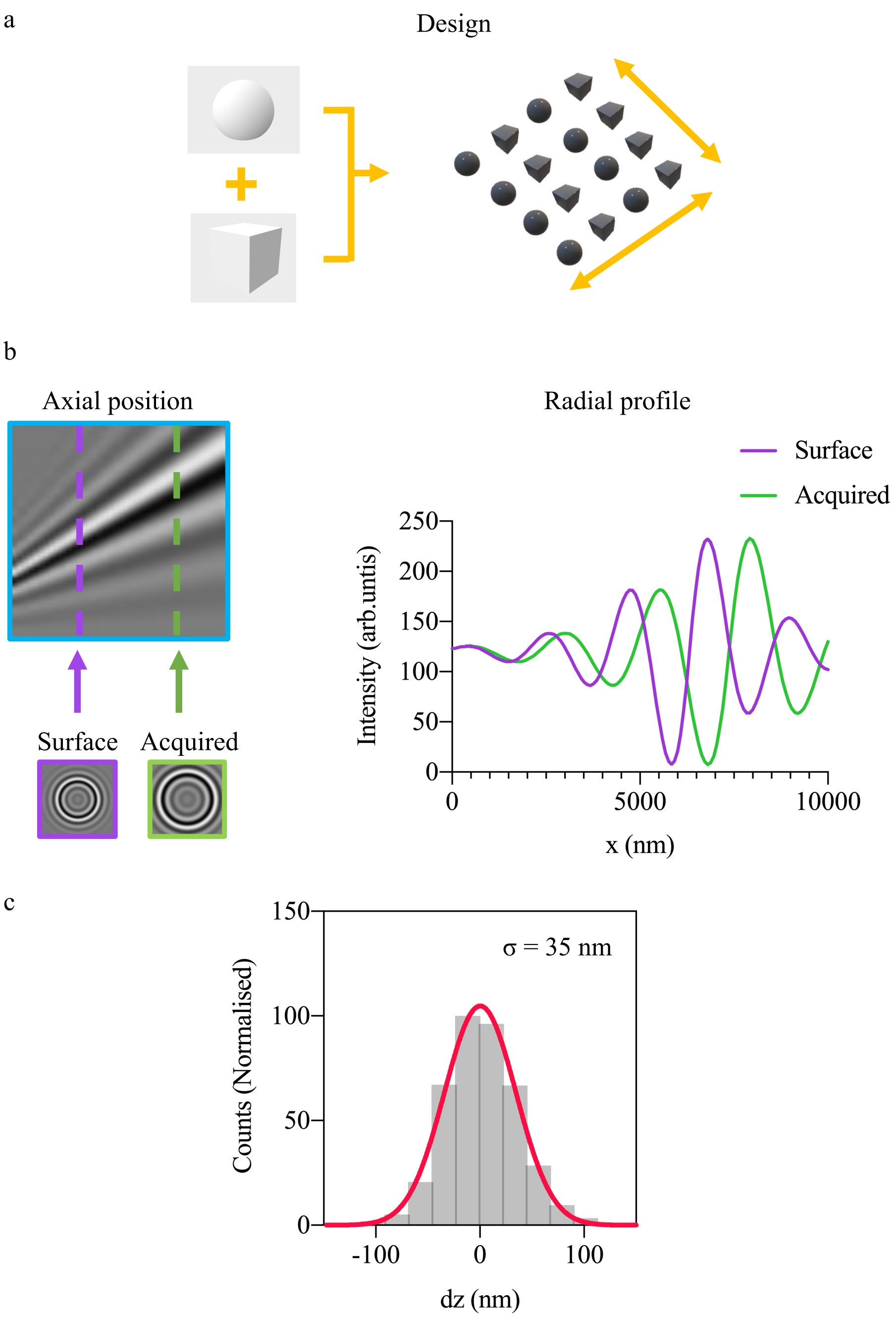

Supplementary Figure 5: Automatic surface detection. **a**, The geometry of the fiducials and their distribution throughout the imaging slide were added to the acquisition software. Fiducials were produced with a regular spacing which matched the field-of-view, thus ensuring at least one per area. The surface was scanned in an *xy* raster pattern and a fiducial was automatically identified once it enters the field-of-view. **b**, Axial offset and surface detection schematic. To determine the *z*-position of the fiducial, the radial profile along the z-axis (simulation in blue box) was acquired and compared to a previously acquired look-up table. To focus on the surface, the axial difference between the surface (purple line) and the acquired position (green line) was determined and the stage was moved. The radial profiles along the z-axis are then re-acquired with continuously smaller axial steps in order to improve accuracy. **c**, Accuracy of the automatic surface detection routine was 35 nm. We deposited individual molecules onto coverslides and recorded the height difference between our autonomous focusing method and the position with maximum signal of the molecules.

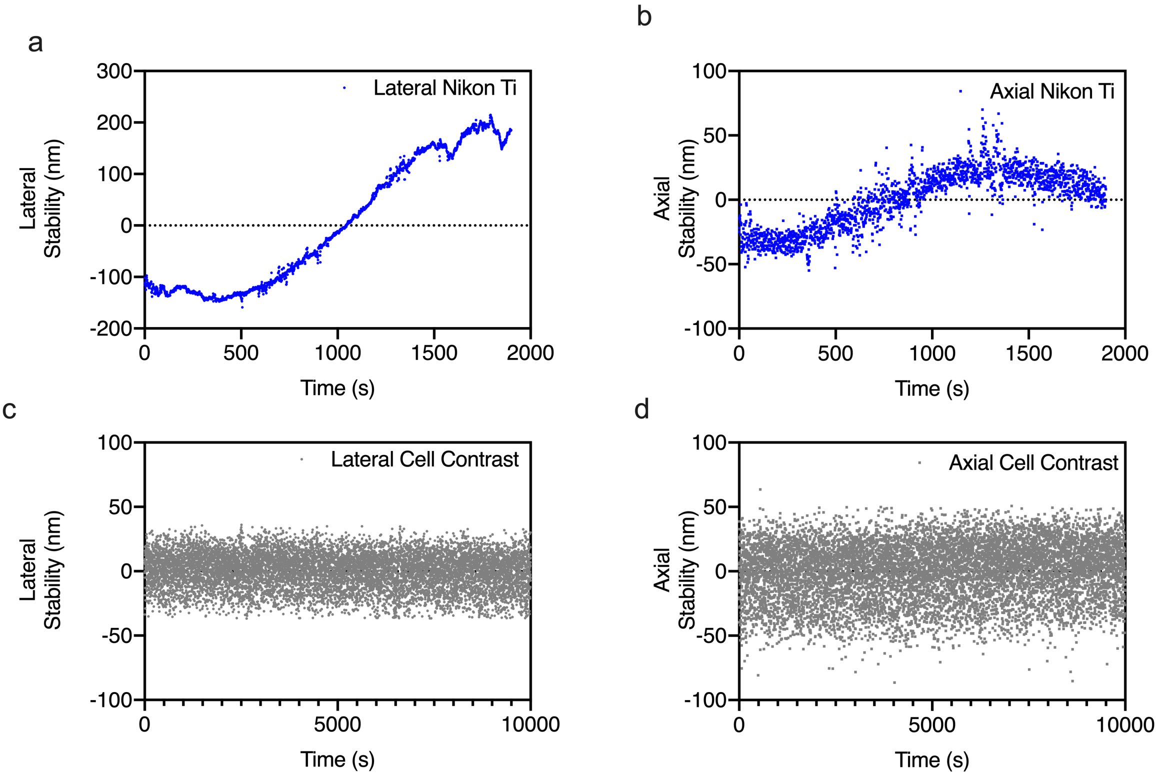

Supplementary Figure 6: 3D focus locking experimental comparison. a-b, Nikon Perfect Focus System. **a**, Lateral (x-direction) and **b**, axial stability of Nikon Perfect Focus determined by monitoring a fiducial in parallel. The standard deviations are 124 nm and 22 nm, respectively. **c-d**, the cell-based image correlation correction. Focus locking was performed using the contrast of cellular structures in three dimensions via image correlation. Drift was monitored in parallel using an out-of-loop fiducial (grey dots, 1/1000 point shown). The standard deviations in lateral **c** and axial **d** directions are 13 nm and 19 nm, respectively. This is comparable to previously published data [1].

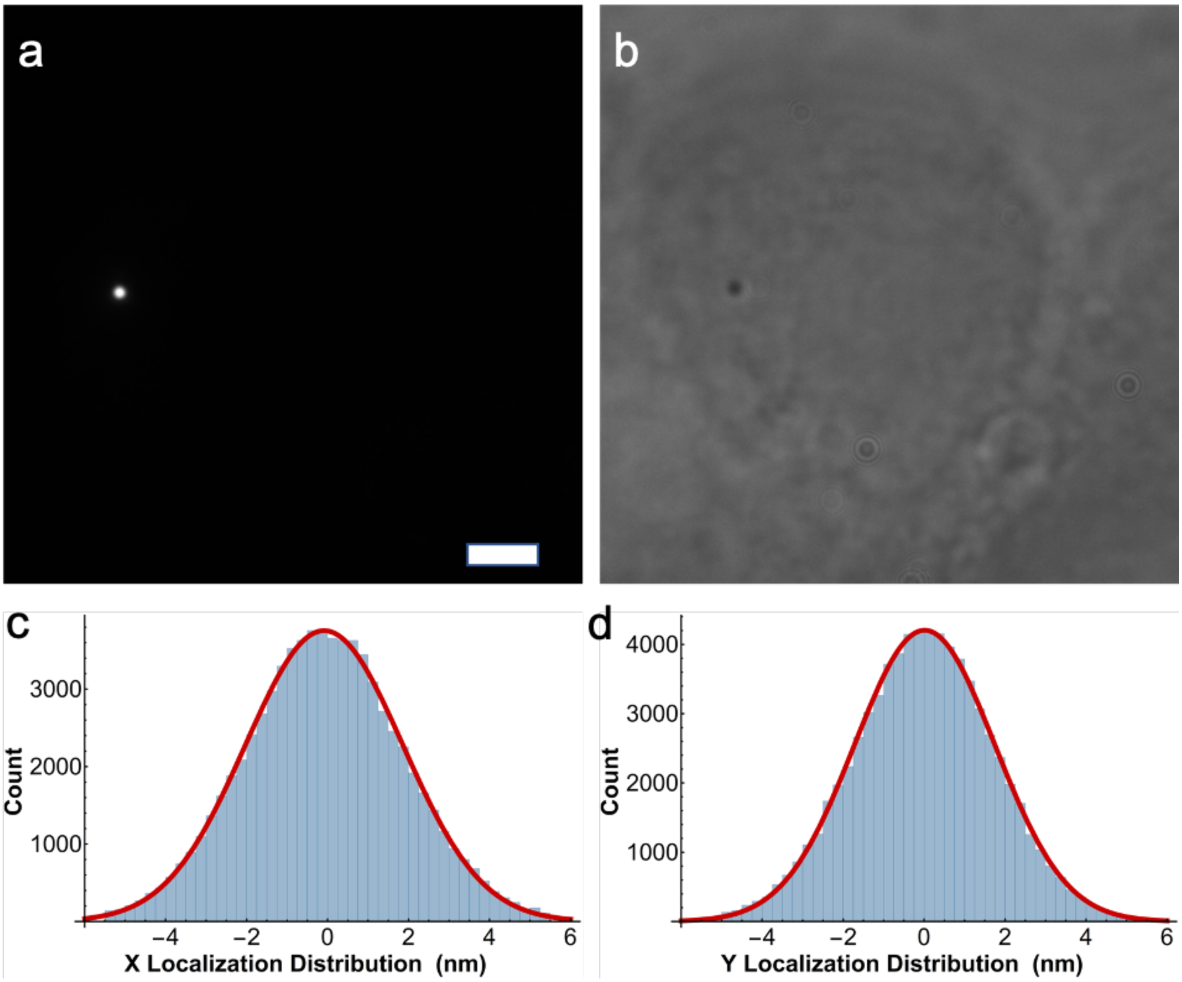

Supplementary Figure 7: Experimental validation of the 3D focus locking. Fluorescent beads were deposited on top of cells. With the 3D focus lock engaged we monitored the position of the fiducial as a function of time. **a,** Widefield image showing a fluorescent bead above a cell at a depth of 5 µm. Scale bar = 3 µm. **b,** Brightfield image of the cell. **c** and **d,** Gaussian fitting of the fiducial indicates a standard deviation of 1.95 nm in the *x* direction and 1.75 nm in the *y* direction after ~ 3 hours. This is consistent with the photon limited localization precision registered, thus demonstrating that the fluorescence remained focused and stable (n = 3).

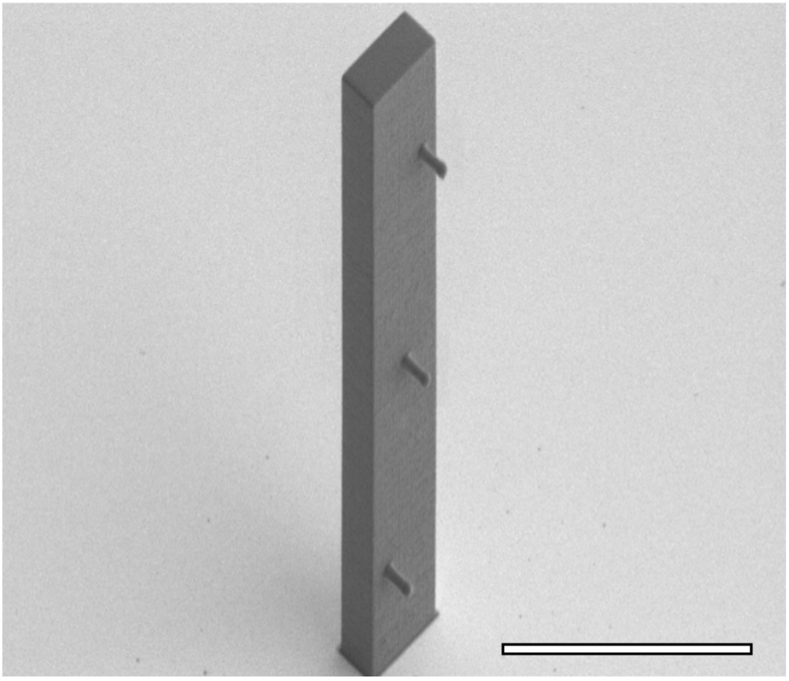

Supplementary Figure 8: Focus locking beyond cellular volumes. Vertically assembled fiducial structures with a height of 100 µm. Scale bar = 50 µm.

**Axial Depth (µm)**	***x* (nm)**	***y* (nm)**	***z* (nm)**
0	0.48	0.57	0.64
1	0.28	0.49	0.68
2	0.49	0.48	0.69
3	0.46	0.46	0.69
4	0.42	0.46	0.74
5	0.53	0.39	0.79
6	0.32	0.44	0.71
7	0.49	0.5	0.74
8	0.53	0.42	0.68
9	0.62	0.45	0.77
10	0.51	0.56	0.69
11	0.39	0.46	0.67
12	0.45	0.51	0.63
13	0.44	0.55	0.77
14	0.48	0.53	0.71
15	0.52	0.5	0.63
16	0.54	0.42	0.8
17	0.41	0.39	0.76
18	0.47	0.56	0.81
19	0.53	0.55	0.79
20	0.6	0.52	0.77
Average	0.47	0.49	0.73

Supplementary Table 1: Sample stability. Standard deviations in the x-, y- and z-direction as a function of depth. The average value was 0.47 nm, 0.49 nm and 0.73 nm for the x-, y- and z-directions, respectively. Focus-locking was performed on nanoprinted pedestals ranging from 0-20 µm. Each depth was acquired for 1 hour, with a combined duration of 21 hours. The standard deviation shown corresponds to out-of-loop reference fiducial acquired simultaneously.

Supplementary References

1. McGorty, R., D. Kamiyama, and B. Huang, *Active microscope stabilization in three dimensions using image correlation.* Optical Nanoscopy, 2013. **2**(1): p. 3.
