# Supplementary material for: Direct-laser writing for subnanometer focusing and single-molecule imaging
Source: Nat Commun. 2022 Feb 3;13:647. doi: 10.1038/s41467-022-28219-6 (PMC8813935; doi:10.1038/s41467-022-28219-6)
Supplement: Supplementary file 3 — Description of Additional Supplementary Files [file 41467_2022_28219_MOESM3_ESM.docx]

**Description of Additional Supplementary Files**

**Supplementary Movie 1:** Real-time subnanometer focusing and dynamic focusing

**Supplementary Movie 2:** Flexibility in Design shown via geometric shapes

**Supplementary Movie 3:** Example of pillars

**Supplementary Movie 4:** Example of pillars enclosed in protection boxes
