# Supplementary material for: Direct-laser writing for subnanometer focusing and single-molecule imaging
Source: Nat Commun. 2022 Feb 3;13:647. doi: 10.1038/s41467-022-28219-6 (PMC8813935; doi:10.1038/s41467-022-28219-6)
Supplement: Supplementary file 8 — Reporting Summary [file 41467_2022_28219_MOESM8_ESM.pdf]

nature research

Corresponding author(s): Simao Coelho, Justin Gooding

Last updated by author(s): Nov 1, 2021

Reporting Summary

Nature Research wishes to improve the reproducibility of the work that we publish. This form provides structure for consistency and transparency in reporting. For further information on Nature Research policies, seeAuthors & Referees and theEditorial Policy Checklist .

Statistics

For all statistical analyses, confirm that the following items are present in the figure legend, table legend, main text, or Methods section.

n/a	Confirmed	
☐	☒	The exact sample size (*n*) for each experimental group/condition, given as a discrete number and unit of measurement
☒	☐	A statement on whether measurements were taken from distinct samples or whether the same sample was measured repeatedly
☒	☐	The statistical test(s) used AND whether they are one- or two-sided<br>*Only common tests should be described solely by name; describe more complex techniques in the Methods section.*
☒	☐	A description of all covariates tested
☐	☒	A description of any assumptions or corrections, such as tests of normality and adjustment for multiple comparisons
☐	☒	A full description of the statistical parameters including central tendency (e.g. means) or other basic estimates (e.g. regression coefficient) AND variation (e.g. standard deviation) or associated estimates of uncertainty (e.g. confidence intervals)
☒	☐	For null hypothesis testing, the test statistic (e.g. *F*, *t*, *r*) with confidence intervals, effect sizes, degrees of freedom and *P* value noted<br>*Give P values as exact values whenever suitable.*
☒	☐	For Bayesian analysis, information on the choice of priors and Markov chain Monte Carlo settings
☒	☐	For hierarchical and complex designs, identification of the appropriate level for tests and full reporting of outcomes
☒	☐	Estimates of effect sizes (e.g. Cohen's *d*, Pearson's *r*), indicating how they were calculated

Our web collection on statistics for biologists contains articles on many of the points above.

Software and code

Policy information about availability of computer code

Data collection	LabVIEW, 2014 64-bit<br>Metamorph 7.10.2.240
Data analysis	ThunderSTORM plugin in ImageJ V1.52 (NIH).<br>TrackMate plugin in ImageJ v3.7 (NIH).<br>Picasso open source software v0.1.0 (Schnitzbauer et al, 2017)<br>Matlab R2019a<br>GraphPad Prism v9

For manuscripts utilizing custom algorithms or software that are central to the research but not yet described in published literature, software must be made available to editors/reviewers. We strongly encourage code deposition in a community repository (e.g. GitHub). See the Nature Research guidelines for submitting code & software for further information.

Data

Policy information about availability of data

All manuscripts must include a data availability statement. This statement should provide the following information, where applicable:

- Accession codes, unique identifiers, or web links for publicly available datasets
- A list of figures that have associated raw data
- A description of any restrictions on data availability

CAD designs, basic explanations on geometries and examples of individual STLs for the nano fabricated fiducials can be found on GitHub (https://github.com/spcoelho/Direct-Laser-Writing-CAD-and-STL). Large SMLM raw data files are available from the corresponding author on reasonable request. Source data is provided with the paper.

Field-specific reporting

Please select the one below that is the best fit for your research. If you are not sure, read the appropriate sections before making your selection.

[x] Life sciences [ ] Behavioural & social sciences [ ] Ecological, evolutionary & environmental sciences

For a reference copy of the document with all sections, see nature.com/documents/nr-reporting-summary-flat.pdf

Life sciences study design

All studies must disclose on these points even when the disclosure is negative.

Sample size	We did not perform sample size calculation as this is a methods paper. However, we ensured sufficient number of samples were collected to demonstrate the usefulness of our new method with always at least three independent acquisitions. Cell samples in the vicinity of fiducial structures were selected for imaging.
Data exclusions	No raw image data was excluded.
Replication	All experiments were replicated successfully. The antibodies used was successfully and reproducibly pretested in individual experiments. Quality control of fiducial structures was performed using scanning electron microscopy and further assessed using brightfield illumination. Experiments were replicated on three occasions independently.
Randomization	Our experiments were concerned with development of direct laser writing and new tools. Thus blinding and randomization is not applicable to our work.
Blinding	Our experiments were concerned with development of direct laser writing and new tools. Thus blinding and randomization is not applicable to our work.

Reporting for specific materials, systems and methods

We require information from authors about some types of materials, experimental systems and methods used in many studies. Here, indicate whether each material, system or method listed is relevant to your study. If you are not sure if a list item applies to your research, read the appropriate section before selecting a response.

Materials & experimental systems

n/a	Involved in the study	
[ ]	[x]	Antibodies
[ ]	[x]	Eukaryotic cell lines
[x]	[ ]	Palaeontology
[x]	[ ]	Animals and other organisms
[x]	[ ]	Human research participants
[x]	[ ]	Clinical data

Methods

n/a	Involved in the study	
[x]	[ ]	ChIP-seq
[x]	[ ]	Flow cytometry
[x]	[ ]	MRI-based neuroimaging

Antibodies

Antibodies used	Anti-alpha-tubulin antibody (DM1A, Sigma-Aldrich)<br>CD47 (Invitrogen, B6512)<br>Alexa Fluor Plus 647 (A32728, Life Technologies)
Validation	All antibodys resulted in specific staining showing the expected structure as expected from manufacturers website. All antibodies were validated by the commercial vendors.

Eukaryotic cell lines

Policy information about cell lines

Cell line source(s)	Cos-7, HEK and HeLa cell lines were purchased from CellBank Australia
Authentication	The cell lines were not authenticated interdependently from CellBank Australia
Mycoplasma contamination	Routine, monthly tests for mycoplasma were conducted on all cell lines used in this study. Cell lines were mycoplasma negative in all screens.

Commonly misidentified lines<br>(See ICLAC register)	No commonly misidentified cell lines were used.
