# Supplementary material for: Direct-laser writing for subnanometer focusing and single-molecule imaging
Source: Nat Commun. 2022 Feb 3;13:647. doi: 10.1038/s41467-022-28219-6 (PMC8813935; doi:10.1038/s41467-022-28219-6)
Supplement: Supplementary file 9 — Related Manuscript File [file 41467_2022_28219_MOESM9_ESM.zip › ImageJ Plugin/Guide.rtf]

There are two files -  a Jar and the parameters file. To install it, dump these two files into the micromanager plugin folder (for me this is C:\Program Files\Micro-Manager-2.0gamma\mmplugins). In micromanager, you then need to have 3 things:1)The camera you want to use installed and running as the primary camera2) The pixel size set correctly for the camera3)the xy and z stages that you want to control installed and set as the Core-XY stage in the device property browser.To run it. in micromanager go Plugins->Feedback Focus lockThe main GUI will then come up.Using the ImageJ panel draw a box over the bead that you want to track then click select bead. If you want to track a second bead to measure errors draw a second box on the live screen then click select second box.Click calibrate to generate the calibration zstack. The correlation plot that is made at the end should look like a smooth inverted parabola. If it is jagged you probably need to increase the delta zslices in the options and rerun calibrate.Click lock and then it should hold the bead in position.When you're finished stop live to stop locking the bead. It should give you an option to save a csv that plots the XYZ position of the stage at each move point as well as the measured position of bead 1 and bead 2.
